# Climate-Driven Distribution and Ecological Niche Modeling of Three *Anopheles* Species in China Using the Biomod2 Ensemble Framework

**DOI:** 10.3390/tropicalmed11070189

**Published:** 2026-07-09

**Authors:** Dan Jiang, Kun Wang, Shenbo Chen, Yang Hong, Senping Yang, Xiaoyuan Su, Yongdong Hao, Fei Luo, Junhu Chen

**Affiliations:** 1 National Key Laboratory of Intelligent Tracking and Forecasting for Infectious Diseases, National Institute of Parasitic Diseases, Chinese Center for Disease Control and Prevention (Chinese Center for Tropical Diseases Research), National Health Commission of the People’s Republic of China (NHC) Key Laboratory of Parasite and Vector Biology, World Health Organization (WHO) Collaborating Center for Tropical Diseases, National Center for International Research on Tropical Diseases, Shanghai 200025, China; 2 Liangjiang New Area Center for Disease Control and Prevention, Chongqing 401122, China; 3 School of Public Health, Hangzhou Medical College, Hangzhou 310013, China; 4 Hainan Tropical Diseases Research Center, Haikou 571199, China; 5 Chongqing Center for Diseases Control and Prevention, Chongqing 401122, China

**Keywords:** *Anopheles*, ensemble model, climate change, suitable habitat

## Abstract

This study aimed to project the current and future suitable habitats of three primary malaria vectors in China—*An. lesteri*, *An. minimus*, and *An. sinensis*—using an ensemble modeling approach. We simulated their geographical distributions under current and future climates (SSP126, SSP245, SSP585) using the Biomod2 platform with 19 bioclimatic variables and elevation. The ensemble models achieved high predictive performance, as reflected by AUC and TSS values. Environmental drivers were species-specific: *An. lesteri* was primarily influenced by elevation, temperature seasonality (Bio4), and seasonal precipitation (Bio18, Bio19); *An. minimus* by the mean temperature of the coldest quarter (Bio11); and *An. sinensis* by annual precipitation (Bio12), mean temperature of the wettest quarter (Bio8), and elevation. Future projections revealed divergent responses: the habitat of *An. lesteri* is projected to contract and shift northeastward; *An. sinensis* is expected to expand northward, potentially extending climatically suitable areas into new regions; and although the overall range of *An. minimus* remains stable, its internal suitability shifts toward higher classes under warming. These findings demonstrate that climate change will critically reshape the distribution of major malaria vectors across China, underscoring the need to integrate climate-informed projections into adaptive surveillance and vector control strategies in the post-elimination era.

## 1. Introduction

After decades of integrated control, China achieved WHO malaria-free certification in 2021 [1]. However, persistent imported malaria cases, coupled with the sustained presence of *Anopheles* vectors across China, maintain the risk of malaria re-establishment in an era of increasing global connectivity. Approximately 450 *Anopheles* species have been documented worldwide, with 60 recorded in China [2]. Among them, *An. sinensis*, *An. lesteri*, *An. minimus*, and *An. dirus* are recognized as the four primary malaria vectors [3]. Their divergent niches, distributions, and vector capacities not only shaped historical malaria transmission dynamics but also underpinned the spatial heterogeneity of re-establishment risk in the post-elimination era. China’s diverse climates, including monsoon in the east, temperate continental in the northwest, and alpine climate on the Qinghai–Tibet Plateau, result in highly uneven precipitation decreasing from southeast to northwest [4]. At the intra-annual scale, rainfall is concentrated in summer and scarce in winter, while temperatures follow a similar seasonal rhythm with warm summers and cold winters. Over the past 50 years, the national annual mean temperature has risen, exceeding the global average. Climate warming, along with human activities such as urbanization and industrial emissions, has also affected total precipitation and its seasonal distribution. These climatic features, together with the annual cycles of rainfall and temperature, thereby shape the spatial distribution of *Anopheles* species across China. Climate change is profoundly altering mosquito distributions and life histories, with cascading effects on transmission potential. Rising temperatures, for instance, accelerate mosquito development and extend their active seasons [5,6], while high humidity further prolongs survival, synergistically enhancing the transmission capacity of vector populations [7,8]. For *An. sinensis*, a rice-field-breeding species, climate-driven shifts may expand its suitable habitats [9]. These changes underscore the need to project future vector dynamics under anticipated climate scenarios to inform proactive surveillance and targeted control.

Grounded in ecological and statistical principles, species distribution models (SDMs) provide a framework for evaluating range shifts under climate change [10]. Early studies used single models like MaxEnt to delineate current suitable habitats [11]. Bayesian Additive Regression Trees (BART), a non-parametric machine learning algorithm, has been increasingly applied in species distribution modeling due to its flexibility and robustness [12]. However, single-model approaches are limited by theoretical assumptions and data sensitivity, introduce high uncertainty and structural dependence in projections. Ensemble modeling, as implemented in Biomod2, combines multiple algorithms to reduce individual biases, thereby improving prediction stability and accuracy [13,14].

In this study, we employed an ensemble modeling strategy within Biomod2 to project the potential distributions of three malaria vectors (*An. sinensis*, *An. lesteri*, *An. minimus*) under current and future climate scenarios (2041–2060, 2061–2080, 2081–2100). Mainly focuses on four core objectives: (1) identifying the dominant environmental factors shaping three *Anopheles* vectors spatial distributions; (2) evaluating current climatic suitability; (3) projecting shifts in future climatically suitable habitat under three Shared Socioeconomic Pathways (SSP126, SSP245, and SSP585); and (4) characterizing changes in spatial extent and geographic centroids of potential ranges. These findings provide a scientific basis for developing evidence-based vector surveillance, early warning systems, and climate-resilient malaria control strategies in China’s post-elimination era.

## 2. Materials and Methods

### 2.1. Occurrence Records

Distribution records of the three *Anopheles* species in China were compiled from the literature published between 1979 and 2025, retrieved from CNKI and PubMed (Supplementary Table S1). Records lacking geographic coordinates were supplemented using Amap (https://lbs.amap.com), and duplicate or erroneous entries were removed in R (v4.5.2) [15,16]. Spatial thinning was applied using the spThin package (1 km distance threshold, 100 iterations) to minimize spatial clustering and reduce spatial autocorrelation (Supplementary Figure S1); pseudo-absence points were randomly generated in three replicates across the study area, with each replicate matching the number of thinned presence points to ensure balanced model training, resulting in a 1:1 presence-to-pseudo-absence ratio.

### 2.2. Environmental Parameters

Bioclimatic and elevation data were sourced from WorldClim version 2.1 at a spatial resolution of 30 arc-seconds [17] (Table 1). The dataset comprised historical climate conditions (1970–2000) and future projections for three periods (2041–2060, 2061–2080, 2081–2100) under three Shared Socioeconomic Pathways (SSP126, SSP245 and SSP585) [18]. Future projections were derived from the CMIP6 BCC-CSM2-MR model, downscaled and bias-corrected using WorldClim v2.1 (1970–2000) as the baseline climate. To mitigate multicollinearity, environmental variables were extracted at occurrence points, and a Spearman correlation coefficient analysis (|r| ≤ 0.8) [19] was applied. Through this procedure, largely independent predictors were selected for subsequent modeling (Supplementary Table S2). The retained variables capture key climatic dimensions influencing mosquito distributions, including temperature-related (Bio2, Bio3, Bio4, Bio5, Bio8) and precipitation-related factors (Bio15, Bio18, Bio19), covering both mean conditions and seasonal and extreme variability critical for mosquito survival and development. Under future scenarios, these variables are projected to shift toward higher temperatures and increased precipitation variability, particularly at higher latitudes and under higher emission scenarios as projected by IPCC AR6. All environmental layers were georeferenced to WGS84 for spatial consistency.

### 2.3. Model Settings and Assessment

By modeling the species–environment relationships of *Anopheles* mosquitoes in China with Biomod2 (v 3.5.1) using R [20], we projected their spatial distributions under current climatic conditions and three future periods: the 2050s (2041–2060), 2070s (2061–2080), and 2090s (2081–2100). These nine algorithms were selected to represent a broad spectrum of modeling approaches, including regression-based methods (GLM, MARS), tree-based methods (CTA, GBM, RF), machine learning techniques (ANN, FDA), presence-only methods (MAXENT), and envelope-based approaches (SRE). This diversity allows the ensemble framework to capture both linear and complex nonlinear species–environment relationships, thereby enhancing predictive robustness and reducing the bias inherent in any single algorithm. All algorithms were implemented with their default parameter settings in BIOMOD2. For model calibration, we used three repeated runs, with 75% of occurrence records randomly selected for training and the remaining 25% for testing [21]. Model performance was evaluated using the True Skill Statistic (TSS) and the area under the ROC curve (AUC-ROC). AUC ranges from 0 to 1, with higher values indicating stronger discriminatory ability, reflecting the capacity to correctly distinguish species presence records from background sites; TSS incorporates both sensitivity and specificity, yielding values from −1 to 1, where values closer to 1 reflect near-perfect prediction accuracy [22,23,24]. Individual models with TSS > 0.7 were retained for ensemble construction combined them using TSS-weighted averaging, giving higher weight to better-performing models. The final ensemble model was assessed using the same metrics. TSS > 0.7 indicates outstanding predictive performance and AUC was interpreted according to the following classification: AUC < 0.6, inadequate; 0.6 ≤ AUC < 0.7, poor; 0.7 ≤ AUC < 0.8, moderate; 0.8 ≤ AUC < 0.9, good; AUC ≥ 0.9, excellent [25].

The ensemble model outputs continuous habitat suitability values ranging from 0 to 1, with higher values indicating greater environmental suitability for the target species. These values were then classified into four categories: unsuitable (0–0.2), low suitability (0.2–0.4), medium suitability (0.4–0.6), and high suitability (0.6–1.0) [19,26]. We converted the continuous suitability values into binary presence-absence layers using the TSS-maximizing threshold. To identify potential expansion pathways, we calculated the geographic centroid of suitable habitat for each future period. These centroid trajectories were mapped in ArcGIS 10.7 [27,28] to illustrate spatiotemporal shifts in distributional centers under changing climatic conditions.

### 2.4. Analysis of the Migration of the Center Point of the Suitable Area

Temporal trends in suitable habitat changes for the three *Anopheles* species were analyzed using the SDM toolbox (version 2.6) [29,30], a Python-based GIS software package, to identify the geometric centers of suitable habitats. Vector layers of overall suitable habitats were generated to visualize centroid shifts, providing quantitative insights into the direction and magnitude of habitat redistribution under varying climatic conditions. Spatiotemporal dynamics were further characterized by tracking centroid movements across different time periods.

## 3. Results

### 3.1. Comparison of Model Performance

**Figure 1:**
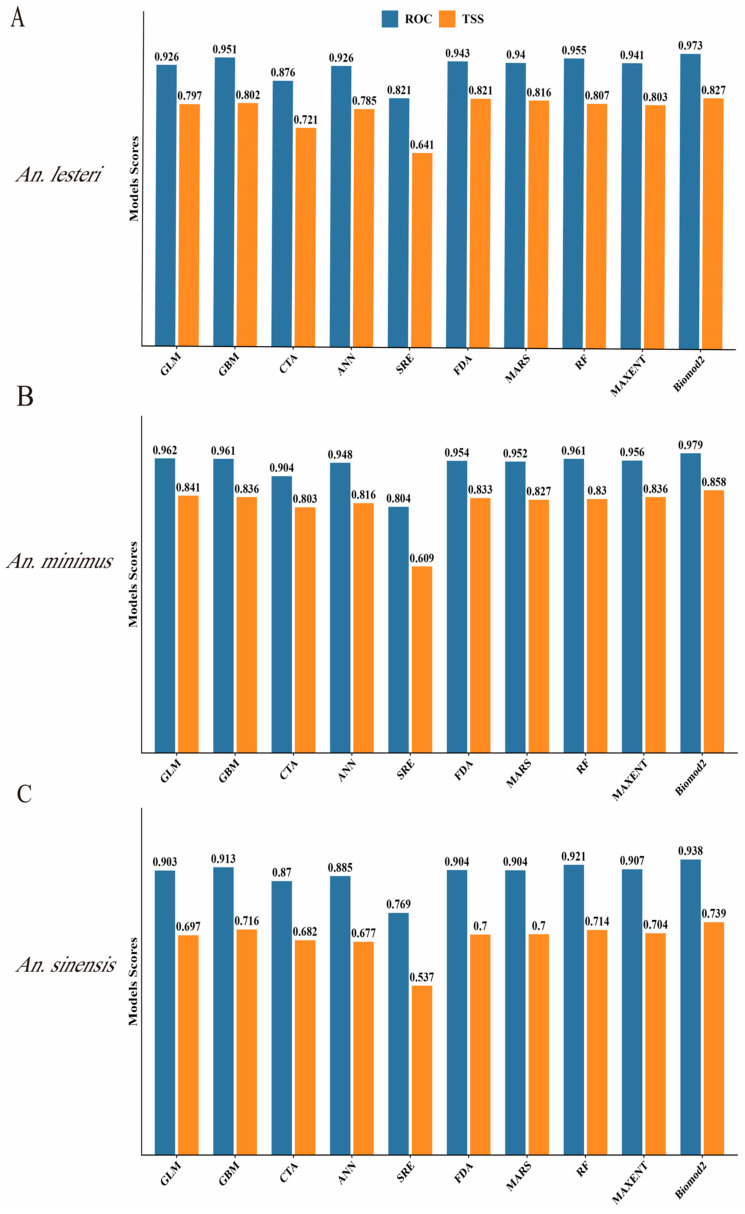
The performance evaluation of individual and ensemble models used for predicting the potential distribution of *An. lesteri* (**A**), *An. minimus* (**B**) and *An. sinensis* (**C**).

The predictive performance of species distribution modeling algorithms was evaluated for three malaria vectors: *An. lesteri*, *An. minimus*, and *An. sinensis*. For *An. lesteri*, strong predictive performance was exhibited by RF and GBM (Figure 1A). In contrast, the SRE model performed poorly, yielding a TSS of only 0.641. For *An. minimus*, the GLM achieved strong predictive power, with ROC and TSS values of 0.962 and 0.841, competitive performance was also observed for GBM (Figure 1B). Regarding *An. sinensis*, the highest ROC and TSS values were again achieved by RF, whereas the weakest performance across metrics was consistently exhibited by SRE (Figure 1C). Across all species, ensemble modeling outperformed all individual algorithms in both ROC and TSS. Therefore, all subsequent habitat suitability assessments were derived from the ensemble model outputs.

### 3.2. Importance of Environmental Factors

**Figure 2:**
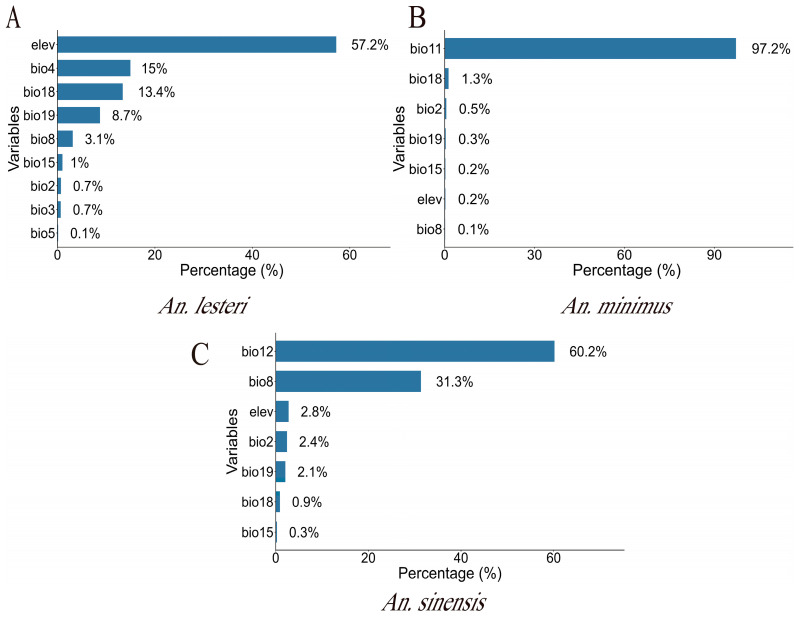
Importance of various environmental variables on three Anopheles species. *An. lesteri* (**A**), *An. minimus* (**B**) and *An. sinensis* (**C**).

Variable importance analysis within the Biomod2 platform identified the dominant environmental factors shaping the geographic distributions of the three *Anopheles* species (Figure 2). For *An. lesteri*, elevation was the strongest predictor, followed by bio4, bio18 and bio19—the four variables with the highest importance scores. For *An. minimus*, bio11 displayed overwhelming importance among all environmental variables. Regarding *An. sinensis*, bio12, bio8 and elevation were identified as the key predictors, forming the primary set of explanatory variables for this species’ distribution.

### 3.3. Environmental Factor Response Curves

**Figure 3:**
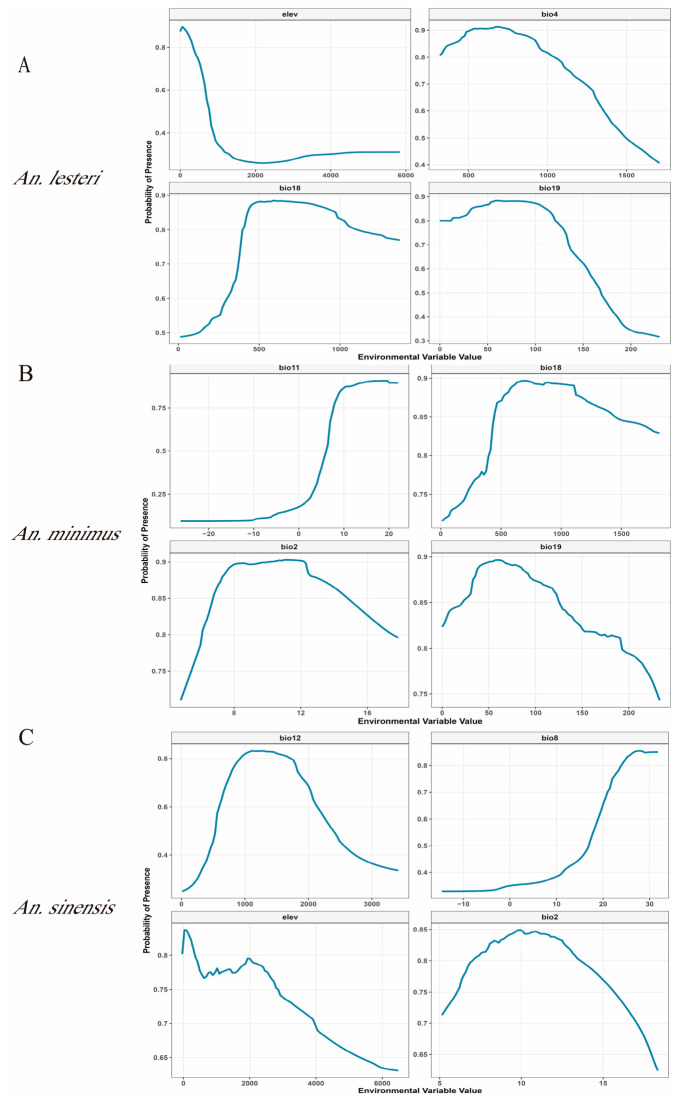
Response curves for dominant environmental variables in the species distribution model for *An. lesteri* (**A**), *An. minimus* (**B**) and *An. sinensis* (**C**) respectively.

For each of the three *Anopheles* species, four critical environmental variables were identified, collectively explaining more than 95% of their respective distribution patterns (Table 1). Response curves derived from the ensemble model further revealed distinct environmental preferences among these vectors (Figure 3). For *An. lesteri*, habitat suitability peaked at 58 m elevation and declined sharply at higher altitudes. Optimal suitability was observed at a Bio4 (temperature seasonality) value of 684.08, a Bio18 (precipitation of the warmest quarter) value of 601.84 mm, and a Bio19 (precipitation of the coldest quarter) value of 60.40 mm (Figure 3A). For *An. minimus*, habitat suitability associated with Bio11 peaked at 19.59 °C before plateauing. In contrast, Bio2, Bio18, and Bio19 each exhibited unimodal responses, indicating optimal suitability at intermediate values (Figure 3B). For *An. sinensis*, response curves revealed distinct optima across environmental variables. Bio12 followed a unimodal distribution, peaking at 1111.96 mm. Bio8 reached a maximum at 27.55 °C before plateauing, whereas Bio2 attained its optimum at 9.94 °C. Elev showed optimal suitability at approximately 31.79 m, with suitability declining progressively at higher altitudes (Figure 3C).

### 3.4. Current Habitats of Three Anopheles Species

**Figure 4:**
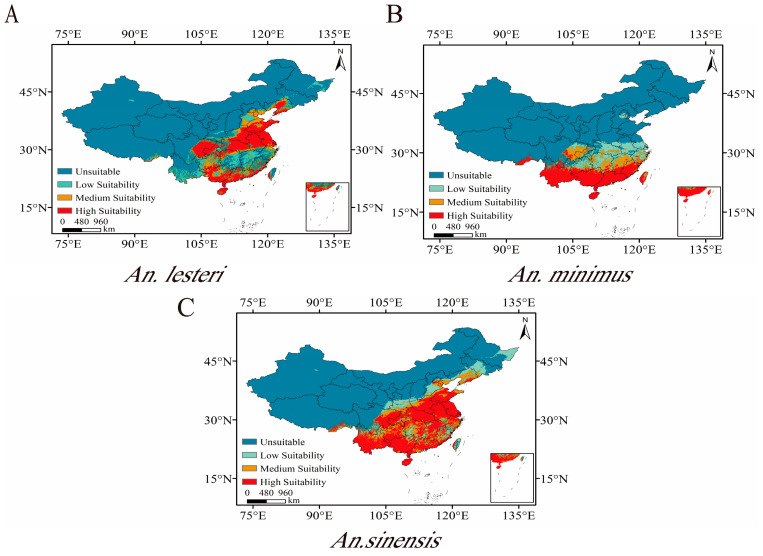
Distribution map of suitable areas in the current period for *An. lesteri* (**A**), *An. minimus* (**B**) and *An. sinensis* (**C**) respectively.

Based on the ensemble projections under current climatic conditions, the three *Anopheles* species exhibited different geographic distributions in China. For *An. lesteri*, the total suitable habitat area was estimated at 2.41 × 10^6^ km^2^, accounting for 25.14% of national territory. High- and medium-suitability zones constituted 10.45% and 5.71% respectively, and were primarily distributed across Central South and East China. In addition, patches of high-suitability habitat were identified in Chongqing, Guizhou, eastern Sichuan, and Liaoning province (Figure 4A). For *An. minimus,* suitable habitats were largely confined to regions south of the Qinling–Huaihe Line, with a total area of approximately 2.00 × 10^6^ km^2^ (20.86% of national territory), high-suitability areas (9.24% of national territory) are concentrated in Yunnan, Guangxi, Guangdong, Hainan, Fujian and Taiwan province, with localized patches also present in southern Sichuan, southern Jiangxi, southern Guizhou, southern Hunan, and southeastern Tibet. Medium- and low-suitability habitats extended inland from these core high-suitability zones (Figure 4B). *An. sinensis* exhibited the most extensive suitable range, estimated at 3.12 × 10^6^ km^2^. A contiguous high-suitability core spanning 1.65 × 10^6^ km^2^ extended northward into provinces such as Henan and Shandong. Additionally, smaller high-suitability patches were identified in Liaoning, Hebei, Beijing, and Tibet (Figure 4C). 

### 3.5. Future Changes in Suitable Habitat Area

**Figure 5:**
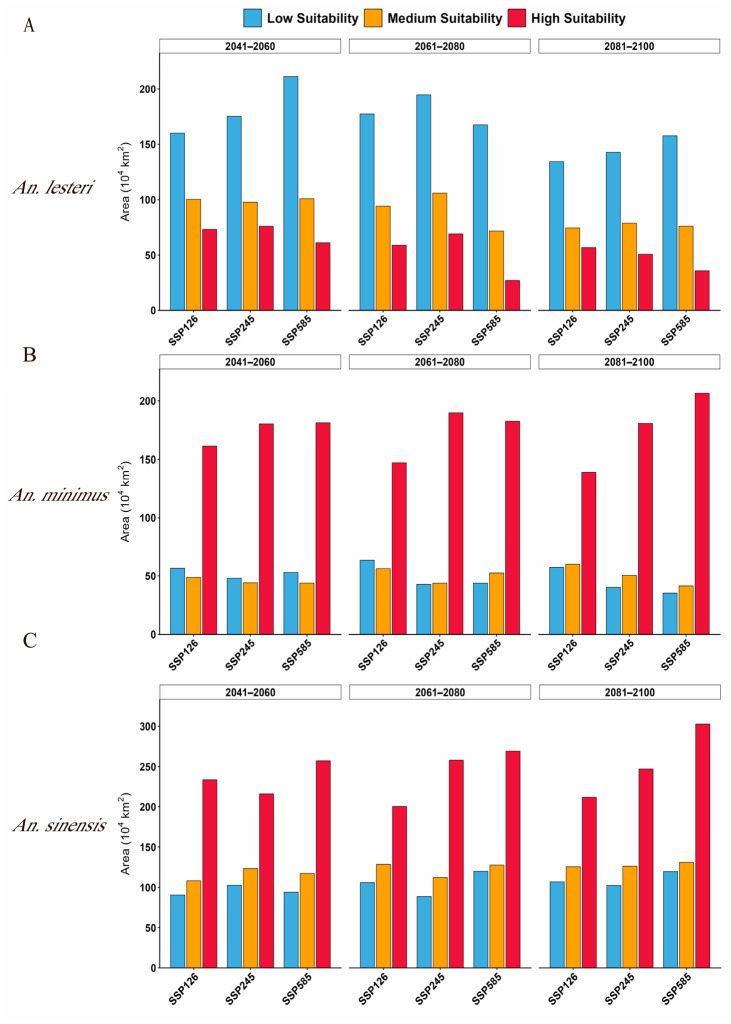
Changes in the suitable habitat area for *An. lesteri* (**A**), *An. minimus* (**B**) and *An. sinensis* (**C**) under different current climatic conditions.

**Figure 6:**
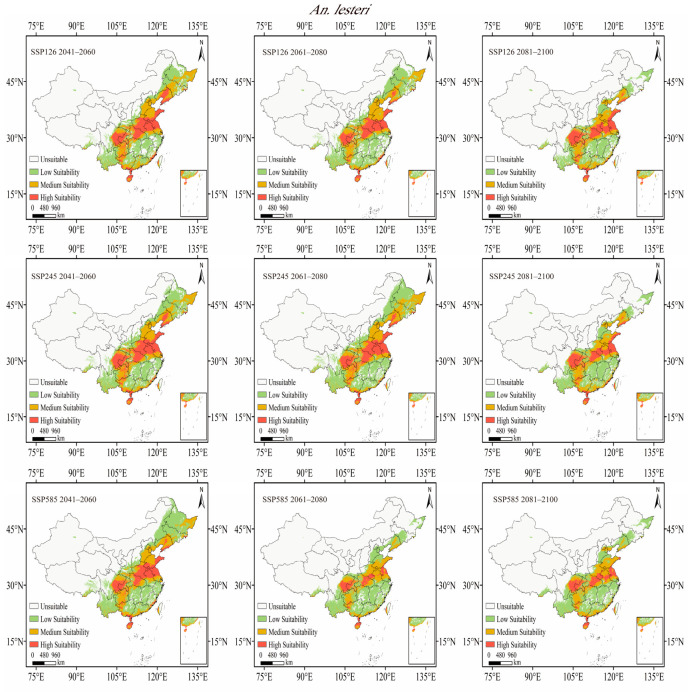
Potential distribution of suitable habitat for *An. lesteri* under future climate conditions.

Compared to the current habitats, total suitable area increased across all future periods (Supplementary Table S3). Under the SSP126 scenario, the total suitable habitat area for *An. lesteri* remained relatively stable from 2041–2060 to 2061–2080, before declining to 2.65 × 10^6^ km^2^ by 2081–2100. The high-suitability area decreased by 22.24% between the 2041–2060 and 2081–2100 periods, while the unsuitable area expanded in the final period. Under SSP245, the total suitable area increased to 3.70 × 10^6^ km^2^ in 2061–2080 before declining to 2.72 × 10^6^ km^2^ by 2081–2100. Regarding habitat structure, medium- and low-suitability areas followed a similar unimodal pattern, rising during 2061–2080 and then falling thereafter. In contrast, the high-suitability area exhibited a consistent decline across all future periods relative to the current period. Under SSP585, the total suitable area peaked at 3.73 × 10^6^ km^2^ in 2041–2060, followed by a sharp decline over the subsequent two periods. Concurrently, the high-suitability area plummeted from 0.61 × 10^6^ km^2^ in 2041–2060 to 0.26 × 10^6^ km^2^ by 2061–2080, reflecting severe habitat degradation under high-emission forcing (Figure 5A). Under SSP126 and SSP245, reductions in high-suitability area relative to current conditions were concentrated in Guangxi, Guangdong, and Shandong, with additional reductions in Liaoning province emerging progressively over time. Medium-suitability habitats expanded in northeastern China during 2041–2060 and 2061–2080, but contracted sharply by 2081–2100. In contrast, under SSP585, suitable areas across all suitable classes declined (Figure 6).

**Figure 7:**
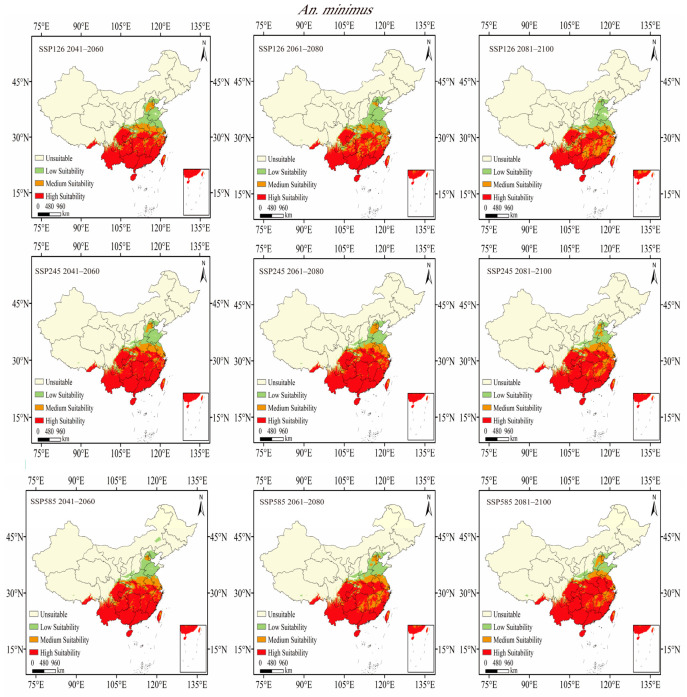
Potential distribution of suitable habitat for *An. minimus* under future climate conditions.

For *An. minimus*, total suitable area remained relatively stable across future scenarios, yet its internal composition shifted markedly. Under SSP126, high-suitability area consistently declined, decreasing by 13.91% between 2041–2060 and 2081–2100, whereas medium-suitability area expanded. SSP245 exhibited minimal change in both total suitable area and suitable proportions. Under SSP585, high-suitability area increased by 13.97% between 2041–2060 and 2081–2100, accompanied by a contraction in low-suitability area, suggesting a shift toward high suitability under intensified climatic stress (Figure 5B). Spatially, compared to the current habitats, suitable habitats remained concentrated south of the Qinling–Huaihe Line. However, medium- and high-suitability zones were projected to expand northward across scenarios and over time. Projected high-suitability gains were observed in Guizhou, Sichuan, Chongqing, Hubei, Hunan, Jiangxi, Zhejiang province, while medium-suitability areas expanded into Jiangsu, Anhui, Henan and Shanghai province, (Figure 7).

**Figure 8:**
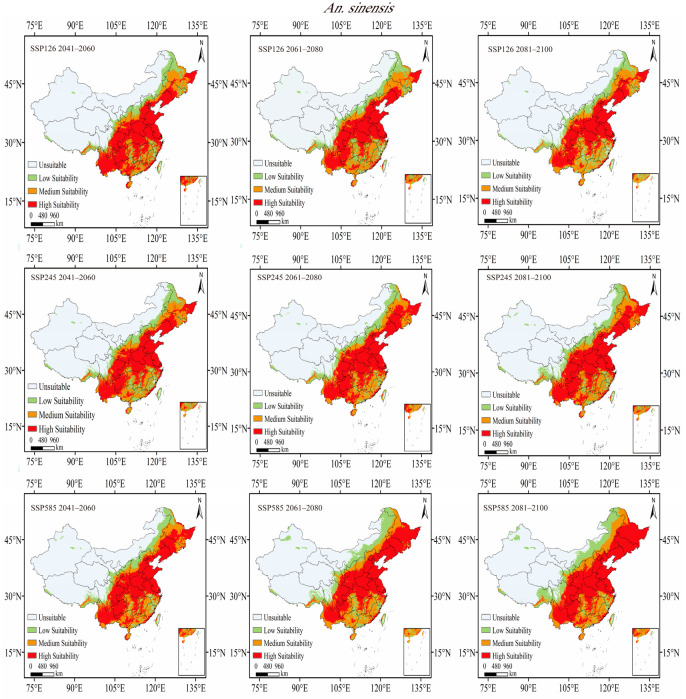
Potential distribution of suitable habitat for *An. sinensis* under future climate conditions.

For *An. sinensis*, total suitable area increased steadily across all three emission scenarios, with high-suitability area consistently remaining above current levels. Under SSP126, high-suitability area contracted during the mid-century period before partially recovering, while medium- and low- suitability areas expanded progressively, indicating a structural shift toward lower-suitability habitats. Spatially, reductions in high-suitability area were observed in Guangdong and Guangxi province, whereas expansions occurred in Heilongjiang province. Under SSP245, total suitable area reached approximately 4.76 × 10^6^ km^2^ by 2081–2100. High-suitability area expanded during 2061–2080 in portions of Guangxi, Hunan, Jiangxi, Zhejiang, Heilongjiang, and Jilin province. By 2081–2100, it declined in Guangxi, Hunan, and Jiangxi province, but continued to increase in Heilongjiang and Jilin province, with sporadic emergence in Inner Mongolia. Meanwhile, medium-suitability area expanded to cover most of northeastern China. The most extensive expansion occurred under SSP585, where total suitable area surged to 5.53 × 10^6^ km^2^ by 2081–2100. High-suitability area peaked at 3.02 × 10^6^ km^2^ during 2061–2080 (Figure 5C). Suitable habitats expanded to cover most of northeastern China, while low-suitability area also increased in parts of Inner Mongolia and Xinjiang (Figure 8).

### 3.6. Centroids Migration of Suitable Areas in the Coming Period

**Figure 9:**
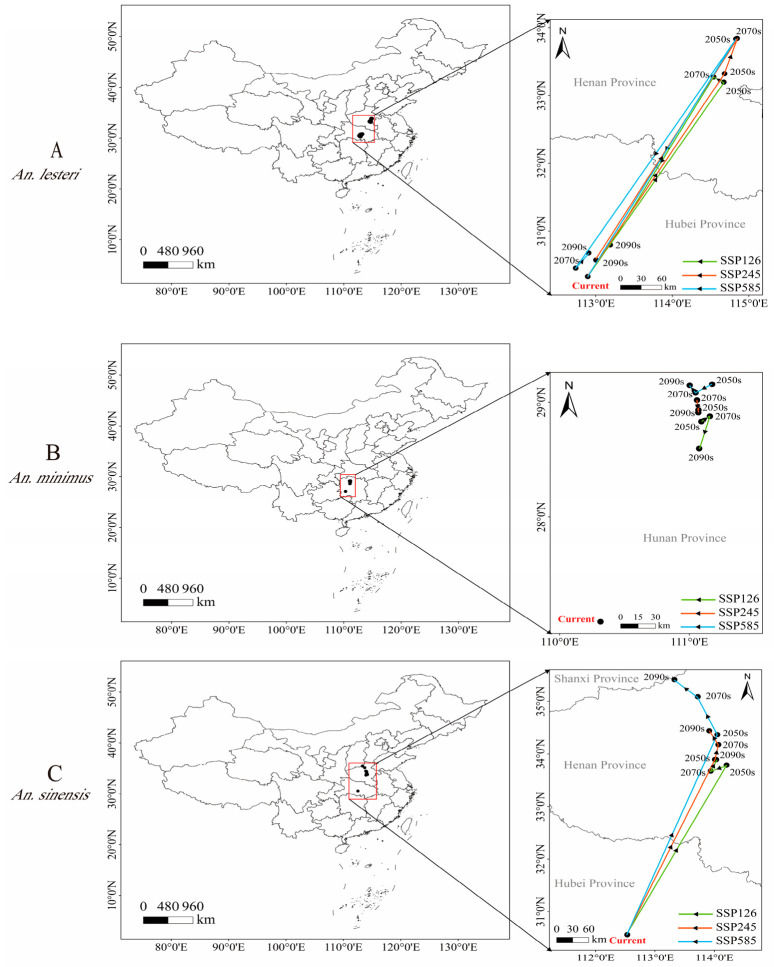
Situation of centroid shift of suitable areas of *An. lesteri* (**A**), *An. minimus* (**B**) and *An. sinensis* (**C**) in different periods.

The spatial centroids of suitable habitats for the three *Anopheles* species were calculated by ArcGIS to quantify their distributional shifts under future climate scenarios. For *An. lesteri*, the current centroid is located in Qianjiang, Hubei province (112.896° E, 30.331° N). Across all scenarios, it shifted northeastward into Henan by 2041–2060 before migrating southward in later periods. Under SSP126, it returned to Tianmen, Hubei province (113.189° E, 30.794° N) by 2081–2100. Under SSP245, during 2061–2080 the object first shifts further north and then migrates back to Tianmen City (113.004° E, 30.571° N) during 2081–2100. Under SSP585, during 2041–2060 it migrates northward to Zhoukou City, Henan province (114.832° E, 33.835° N), and subsequently re-enters Hubei during 2061–2080 (Figure 9A). The current centroid of *An. minimus* is situated in Shaoyang, Hunan province (110.315° E, 27.087° N). Under SSP126, SSP245, and SSP585, its position remained relatively stable throughout the study period, showing only a gradual, limited northeastward shift within Hunan (Figure 9B). For *An. sinensis*, the present centroid lies in Jinmen, Hubei province (112.534° E, 30.563° N). Under all scenarios, the centroid shifted into Henan during 2041–2060. Under SSP245, the centroid continued migrating northward, reaching Zhengzhou, Henan (113.913° E, 34.440° N) by 2081–2100, whereas under SSP585, it moved further northwest during the same period to Jiaozuo, Henan (113.329° E, 35.411° N) (Figure 9C).

## 4. Discussion

The three *Anopheles* species are the primary malaria vectors in China and play a pivotal role in shaping the spatiotemporal dynamics of malaria transmission. However, most previous studies have relied on single-model approaches, and a systematic ensemble modeling framework for predicting habitat shifts among multiple *Anopheles* species under future climate scenarios remains lacking. By applying an ensemble modeling approach (Biomod2), this study assessed current and projected suitable habitats for three major *Anopheles* species across China. The findings elucidate distinct distributional dynamics and pronounced spatial heterogeneity under climate change, offering a scientific foundation for long-term risk early warning systems and the development of targeted, species-specific control strategies.

### 4.1. Model Performance and Reliability Analysis

Although individual algorithms performed well for specific species, with RF and GBM for *An. lesteri* and *An. sinensis*, and GLM for *An. minimus*, reliance on any single model may limit projection reliability and practical utility [31,32]. Our results show that the ensemble model consistently outperformed all individual algorithms (including MaxEnt) across the three *Anopheles* species, based on both ROC and TSS metrics (Figure 1). These results confirm that ensemble forecasting reduces the biases inherent in single-algorithm approaches, improving the accuracy and robustness of distribution projections. This provides a robust basis for subsequent spatiotemporal analyses under climate change and aligns with previous evidence that ensemble methods improve predictive confidence in ecological niche modeling [33,34].

### 4.2. Ecological Interpretation of Key Environmental Drivers

Climatic conditions—particularly temperature, precipitation, and elevation—directly and indirectly influence vector-borne disease transmission by modulating vector reproduction, development, behavior, and population dynamics, thereby shaping the spatiotemporal distribution of disease risk [35,36]. Elevation, seasonal precipitation (Bio18, Bio19), and temperature variability (Bio4) play crucial roles in the distribution of *An. lesteri* (Figure 2A), indicating that its survival and reproduction are highly dependent on these factors. This species prefers moderate precipitation, a narrow annual temperature range, and lower elevations—conditions consistent with its current range and its known breeding sites (e.g., rice paddies, ponds) [37,38]. In contrast, the distribution of *An. minimus* is overwhelmingly governed by the mean temperature of the coldest quarter (Bio11), which contributed the predominant proportion of variable importance (Figure 2B). This pronounced thermal limitation aligns with its current confinement south of the Qinling–Huaihe Line, coinciding with the subtropical and temperate monsoon zones where the vector is concentrated. It further implies potential northward expansion under projected warming as cold constraints diminish. *An. sinensis* exhibits broader climatic tolerance, shaped jointly by annual precipitation (Bio12), mean temperature of the wettest quarter (Bio8), and elevation (Figure 2C). These environmental drivers not only shape species distributions but also carry different implications for vector control and disease transmission across China. In historically endemic regions of southern China, where vectors are already established, climate factors primarily modulate transmission intensity and seasonality. In contrast, higher altitudes reduce air density, humidity, and temperature—conditions unfavorable for *Anopheles* growth and reproduction [39]. Under future warming, relaxation of these thermal constraints may facilitate northward expansion of *An. sinensis* (and to a lesser extent *An. minimus*) into historically unsuitable areas, potentially increasing the risk of malaria re-establishment in previously low-burden regions of China. These patterns highlights that the public health implications of our projections are regionally differentiated.

### 4.3. Validation and Significance of Current Potential Distribution Patterns

The simulated current distributions of the three *Anopheles* species correspond well with historical records and field surveillance data [40,41], validating model robustness. For *An. lesteri*, high-suitability habitats are concentrated in central and eastern China. Notably, only northern Hunan is classified as medium-to-high suitability, while most of Hunan and adjacent regions (e.g., Jiangxi, Zhejiang) are low suitability—consistent with prior MaxEnt-based results [9] but diverging from BART-based projections [12]. The discrepancy likely stems from differences in algorithm sensitivity to collinearity and interaction effects: BART captures complex nonlinear relationships more flexibly than MaxEnt, whereas our ensemble approach reduces single-model bias through averaging. These methodological contrasts underscore the importance of algorithm choice, particularly in regions with sparse occurrence data or weakly defined species–environment relationships. Beyond algorithm-driven differences, divergences among studies may also reflect natural constraints (elevation, topography, precipitation) and sustained intensive control measures (e.g., indoor residual spraying, insecticide-treated nets, larval source management) [3,42,43,44], all of which collectively shape local distribution patterns. Moreover, field-based manual surveillance is weather-constrained and may underestimate actual distributions. The scattered low-suitability patches thus reflect the interplay of ecological limits and long-term human intervention. *An. minimus* remains largely confined to south of the Qinling–Huaihe Line, with its distribution primarily controlled by winter mean temperature (Bio11)—tracking low-temperature limits rather than high-temperature upper limits (Figure 3B). This implies that under climate warming, northern regions may meet overwintering requirements, potentially providing more favorable conditions for the vector. Nevertheless, these factors also explain the current absence of suitable habitats in northern China despite adequate precipitation and vegetation. Epidemiologically, high-suitability zones substantially overlap with border areas adjacent to Southeast Asia [44], where imported malaria cases remain frequent [45,46,47], which highlights the need for integrated surveillance targeting both local vectors and imported infections. In addition, isolated high-suitability patches in southern Sichuan and southeastern Tibet suggest that topographically sheltered microclimates (e.g., deep river valleys) create locally temperate conditions favorable for *An. minimus*. *An. sinensis* is primarily distributed in warm, humid subtropical and tropical monsoon climates. Its distribution is shaped by moderate annual precipitation, warm wet-quarter temperatures, low elevation, and intermediate diurnal temperature ranges—ecological traits that favor lentic and slow-flowing aquatic habitats typical of lowland agricultural landscapes [48], which in turn provide essential conditions for larval development. These characteristics explain its extensive high-suitability core across the Yangtze Plain, Sichuan Basin, and southern Huang–Huai–Hai Plain.

### 4.4. Responses of Anopheles Mosquitoes to Future Climate Change

*An. lesteri* is projected to experience high-suitability habitat contraction in southern provinces (e.g., Guangxi, Guangdong) as well as eastern and northeastern regions (e.g., Shandong, Liaoning). This pattern is largely attributable to its sensitivity to temperature seasonality. In southern China, projected warming may push conditions beyond the species’ optimum; in northern China, increased temperature and precipitation may create newly suitable environments elsewhere. However, suitable reduction likely results from increased annual temperature range, altered seasonal transitions, heightened precipitation seasonality, and extreme rainfall risks—factors that collectively exceed the species’ climatic tolerance in Liaoning (Figure 6). Thus, *An. lesteri* is subject to multifaceted, latitudinally variable climatic constraints, reflecting strong spatial heterogeneity in its climate response. Under SSP245, the expansion of medium-suitability areas in northeastern China during 2041–2080 created a northward pull on the centroid. However, by 2081–2100, the substantial loss of high-suitability areas in Henan, combined with the reduction in medium-suitability areas in the northeast, eliminated this pull and caused the centroid to shift back to its present location near Tianmen, Hubei. Under SSP585, during 2061–2100 the continued reduction in suitable areas in the northeast kept the centroid relatively stable near its current position throughout the entire study period. As a stream-breeding species with narrow habitat requirements, *An. minimus* is projected to maintain a stable distribution under future scenarios, with only minor changes in total suitable area and centroid position (Figure 7 and Figure 9), high-suitability area expands over time across scenarios, suggesting that moderate warming would enhances suitability within the existing range. The projected northward shift of suitable habitats would extend climatically suitable areas into historically low-burden northern provinces, given their limited recent experience in malaria prevention and control. Strengthening capacities in imported case identification, vector surveillance, and intervention responses would therefore be a necessary and prudent preparatory measure. *An. sinensis* exhibited high-suitability habitats concentrated in southern and southwestern China, while medium- and low-suitability zones extended into northeastern provinces, Inner Mongolia, and Xinjiang. This geographic redistribution corresponds with a marked northwestward centroid shift, indicating that climate warming may generate newly suitable habitats in previously cooler regions. Under SSP245, the centroid shifts northeastward to Zhengzhou, Henan; under SSP585, further expansion of suitable areas in Inner Mongolia and the northeast pushes it northwestward to Jiaozuo, Henan by 2081–2100. The species’ strong climate-tracking capacity and broad ecological niche explain its sustained total area expansion across all scenarios. From a control perspective, this northward expansion could introduce vector invasion risks in traditionally low-risk regions (e.g., Northeast China and Inner Mongolia), underscoring the need for enhanced surveillance and risk assessment [49].

### 4.5. Limitations and Future Perspectives

This study has several important limitations that should be acknowledged. Our ensemble modeling framework primarily focused on climatic variables, while other factors, including local environmental conditions (e.g., land use, water body distribution, and ground-level water resources), socioeconomic factors (e.g., population density and urbanization), and ongoing vector control interventions, were not incorporated. In addition, reliable, spatially explicit, and nationally consistent long-term data on vector control activities are currently unavailable, which precluded their integration into the present modeling framework. Occurrence data were obtained solely from the published literature, which may not capture the full geographic range of the three species and may thus underestimate their suitable habitats. Furthermore, the modeling extent was restricted to China, which may not fully account for cross-border dispersal dynamics, particularly in southern border regions where population exchange with neighboring countries could influence local distribution patterns.

A further limitation is that only three SSP scenarios (SSP126, SSP245, and SSP585) were included. Although these represent low-, medium-, and high-emission pathways, the exclusion of intermediate scenarios such as SSP370 and SSP460 may limit the exploration of additional climate–society interaction pathways that could influence vector habitat dynamics. Another limitation concerns the temporal alignment between occurrence records (1979–2025) and the current climate baseline (1970–2000). Records collected after 2000 may deviate from the baseline climate and introduce uncertainty into model calibration. However, this influence is likely to be limited, as broad climatic gradients across the study region are the primary drivers of species distributions. Interspecific interactions and adaptive evolutionary responses to climate change were also not considered. Additionally, the random train–test split may yield optimistic performance metrics (e.g., AUC, TSS). Although we applied 1 km spatial thinning to partially reduce spatial clustering, spatially structured validation would have provided more conservative estimates. Future research that integrates these multidimensional drivers—alongside dynamically updated climate data, spatially explicit field surveillance, and occurrence records from neighboring countries—would substantially enhance the ecological realism, predictive robustness, and practical utility of habitat suitability projections, thereby supporting the design of targeted, adaptive vector management strategies under a warming climate.

## 5. Conclusions

Using ensemble species distribution modeling, we projected current and future potential distributions of three major malaria vectors in China under climate scenarios. Currently, *An. lesteri* is concentrated in southern and eastern China; An. minimus is largely restricted to areas south of the Qinling–Huaihe Line; and *An. sinensis* occupies extensive high-suitability zones across most of southern China. Under future climates, *An. lesteri* shows a pronounced contraction of high-suitability habitats over time across emission scenarios, while its medium- and low-suitability habitats shift northward. *An. sinensis* expands northward and northwestward, extending climatically suitable areas into northeastern China. In contrast, *An. minimus* maintains stable range limits but undergoes internal “quality upgrading,” with high-suitability area expanding under warming. These projections underscore that climate change will likely reshape the distribution of key malaria vectors in China. Integrating climate-informed forecasts into adaptive surveillance and control strategies is therefore essential for targeted interventions in the post-elimination era.

## Figures and Tables

**Table 1:** Environmental variables used in this study.

Category	Environmental Variable	Unit	*An. lesteri*		*An. minimus*		*An. sinensis*	
			Participating in Modeling	% Contribution	Participating in Modeling	% Contribution	Participating in Modeling	% Contribution
Bioclimatic	Annual mean air temperature(Bio1)	°C						
	Mean diurnal range (Mean of monthly (max temp¨Cmin temp)) (Bio2)	°C	√	0.717	√	0.543	√	2.434
	Isothermality (Bio2/Bio7) × 100 (Bio3)	Unitless	√	0.667				
	Variation in temperature seasonality (Bio4)	SD ×100	√	14.977				
	Max temperature of warmest month (Bio5)	°C	√	0.144				
	Min temperature of coldest month (Bio6)	°C						
	Temperature annual range (BIO5-CBIO6) (Bio7)	°C						
	Mean temperature of wettest quarter (Bio8)	°C	√	3.136	√	0.138	√	31.348
	Mean temperature of driest quarter (Bio9)	°C						
	Mean temperature of warmest quarter (Bio10)	°C						
	Mean temperature of coldest quarter (Bio11)	°C			√	97.198		
	Annual precipitation (Bio12)	mm					√	60.174
	Precipitation of wettest month (Bio13)	mm						
	Precipitation of driest month (Bio14)	mm						
	Precipitation seasonality (Coefficient of variation) (Bio15)	Unitless	√	1.023	√	0.239	√	0.265
	Precipitation of wettest quarter (Bio16)	mm						
	Precipitation of driest quarter (Bio17)	mm						
	Precipitation of warmest quarter (Bio18)	mm	√	13.396	√	1.307	√	0.909
	Precipitation of coldest quarter (Bio19)	mm	√	8.722	√	0.339	√	2.08
Topographic	Elevation (elev)	M	√	57.219	√	0.237	√	2.79

**√ denotes that the environmental variable was incorporated into the final ensemble model.**

## Data Availability

The environmental data used in this study are publicly available from the WorldClim database (https://www.worldclim.org). No new data were generated during the study.

## Supplementary Materials

The following supporting information can be downloaded at: https://www.mdpi.com/article/10.3390/tropicalmed11070189/s1, Figure S1: Distribution points of three *Anopheles* species. (A) *An. lesteri*, (B) *An. minimus*, (C) *An. sinensis*. Table S1: Distribution of recorded occurrence points for *Anopheles* species. Table S2: Correlation coefficient matrix of 19 bioclimatic and elev factors. Table S3. Suitable habitat areas (×10^4^ km^2^) for each *Anopheles* species under current and future scenarios across three future periods (2041–2060, 2061–2080, 2081–2100).

## Abbreviations

*An. sinensis*: *Anopheles sinensis*; *An. lesteri*: *Anopheles lesteri*; *An. minimus*: *Anopheles minimus*; *An. dirus*: *Anopheles dirus*; BART: Bayesian Additive Regression Trees; SDMs: Species Distribution Models; CNKI: China National Knowledge Infrastructure; GLM: Generalized Linear Model; GBM: Generalized Boosted Model; CTA: Classification Tree Analysis; ANN: Artificial Neural Network; SRE: Surface Range Envelope; FDA: Flexible Discriminant Analysis; MARS: Multivariate Adaptive Regression Splines; RF: Random Forest; Elev: Elevation; ROC: Receiver Operating Characteristic; TSS: True Skill Statistic.
