# Supplementary material for: Climate-Driven Distribution and Ecological Niche Modeling of Three Anopheles Species in China Using the Biomod2 Ensemble Framework
Source: Trop Med Infect Dis. 2026 Jul 9;11(7):189. doi: 10.3390/tropicalmed11070189 (PMC13419158; doi:10.3390/tropicalmed11070189)
Supplement: Supplementary file 1 [file tropicalmed-11-00189-s001.zip › Supplementary Table S3.pdf]

Supplementary Table S3. Suitable habitat areas (×10⁴ km²) for each *Anopheles* species under current and future climate scenarios, categorized by suitability level (low, medium, and high), across three future periods (2041–2060, 2061–2080, 2081–2100).

	An. lesteri				*An. minimus*				*An. sinensis*			
	Not Suitable	Low Suitable	Medium Suitable	High Suitable	Not Suitable	Low Suitable	Medium Suitable	High Suitable	Not Suitable	Low Suitable	Medium Suitable	High Suitable
Current	719.3063	86.1101	54.86306	100.3916	760.4514	71.22833	40.32889	88.662	647.89	72.845	74.3026	165.63
SSP126 2041-2060	626.9581	160.107	100.3585	73.24792	693.9896	56.68167	48.63437	161.36	528.05	90.692	108.442	233.49
SSP126 2061-2080	629.9752	177.354	94.37556	58.96667	693.5378	63.69792	56.25139	147.15	525.12	106.3	128.816	200.43
SSP126 2081-2100	694.76	134.376	74.58111	56.95403	704.2877	57.37875	60.05333	138.92	515.92	107.08	125.864	211.81
SSP245 2041-2060	611.4806	175.271	97.87333	76.04569	688.2122	47.99021	44.06035	180.4	518.14	102.9	123.676	215.96
SSP245 2061-2080	590.4257	194.806	106.1467	69.29271	684.2581	42.76604	43.80625	189.82	501.25	88.881	112.484	258.05
SSP245 2081-2100	688.3407	142.822	78.76125	50.74757	689.2192	40.33139	50.24833	180.87	484.53	102.74	126.458	246.93
SSP585 2041-2060	587.3313	211.188	100.9673	61.18423	682.3814	53.03923	43.8791	181.35	491.86	94.19	117.393	257.23
SSP585 2061-2080	694.1902	167.699	71.78597	26.99611	681.4886	43.79549	52.65049	182.72	443.4	120.34	127.95	268.98
SSP585 2081-2100	691.0284	157.743	76.10847	35.79118	676.9448	35.56528	41.46861	206.69	406.83	120	131.158	302.68

*Values represent total suitable area summed across not, low, medium, and high suitability categories.
